# Surgery

**Published:** 1900-12

**Authors:** James Swain


### SURGERY.

Many surgeons have discarded the use of **carbolic acid** except for the immersion of instruments which are tarnished by solutions of mercury, but among the public at large, and even some surgeons it is not sufficiently widely known that this too popular antiseptic is liable to cause **gangrene** when applied to the extremities even in dilute solutions. Indeed the dilute solutions—water will practically only dissolve about five per cent.—are the more dangerous because they cause no pain, and their action is therefore more insidious.

My attention has recently been called to this matter by the necessity for amputating a finger, which after an injury at the extremity had been wrapped in a solution of carbolic acid for twenty-four hours and became gangrenous as far up as the first inter-phalangeal joint; and a paper on **carbolic gangrene**, by Dr. Harrington,[1] is a timely reminder that hundreds of fingers have been destroyed from this cause. He has collected a total of one hundred and thirty-two cases of gangrene from dilute solutions of carbolic acid.

It would appear from his observations that any solution of carbolic acid between 1 and 5 per cent. is dangerous, though it is probable that the strength of the solution has less to do with the unfortunate result than the duration of the application, and the thickness of the individual's epidermis. Indeed, he quotes Lévai as showing that strong carbolic acid is less dangerous,

[1] *Am. J. M. Sc.*, 1900, CXX. 1.

for it forms a scab which resists penetration of the acid into the deeper tissues, so that complete gangrene and destruction of an extremity are less likely to occur from the pure liquid acid than from dilute solutions of it.

According to this same observer, the death of the part is due to a direct chemical action on all the tissues. Carbolic acid has no specific quality for the production of the gangrene, for a like effect is produced by five per cent. solutions of hydrochloric, nitric, sulphuric, and acetic acids, and of caustic potash when applied to an extremity by a moistened compress for about twenty-four hours.

Tight bandaging undoubtedly increases the tendency to this process, but experiments have shown that the gangrene does not result primarily from this cause. The treatment of this condition varies according to the severity of the process. If it seems superficial, and the case is seen soon after the removal of the carbolic dressing, it might be beneficial to apply a dressing saturated with lime water, but in other cases it soon becomes evident that amputation is the only resource.

The best prophylactic consists in the avoidance of the use of carbolic acid for wounds, and it is the duty of medical men to show by their example that the public should not make use of this antiseptic.

A subject of far-reaching importance is the value which should be attached to the **use of X-rays in surgery.** Patients not infrequently obtain skiagrams of their lesions, and are frequently surprised to find that, in the case of fractures, the apparent position of the fragments leaves much to be desired, and should an action for malpraxis be instituted, such a skiagram would be likely to appeal to the jury in a way that would be adverse to the medical attendant and the profession at large.

The discrepancy between the skiagram and the lesion as the result of treatment has been commented upon[1] from time to time, but it is well for us all to know exactly where we stand, and to this end I cannot do better than epitomise the conclusions on this subject which were unanimously adopted as expressing the views of the American Surgical Association:[2] views which those who are familiar with X-ray work will fully endorse:—1. The routine employment of the X-ray in cases of fracture is not at present of sufficient definite advantage to justify the teaching that it should be used in every case. If the surgeon is in doubt as to his diagnosis he should make use of this, as of every other available means to add to his knowledge of the case, but even then he should not forget the grave possibilities of misinterpretation. 2. In the regions of the base of the skull, the spine, the

[1] *Brit. M. J.*, 1900, i. 945. *Med. News*, 1900, lxxvi. 192. *Practitioner*, 1899, lxii. 535.
[2] *Am. J. M. Sc.*, 1900, cxx. 35.

pelvis, and the hips, the X-ray results have not as yet been thoroughly satisfactory, although good skiagrams have been made of lesions in the last three localities. 3. As to questions of deformity, skiagrams alone, without expert surgical interpretation, are generally useless and frequently misleading. The appearance of deformity may be produced in any normal bone, and existing deformity may be grossly exaggerated. 4. It is not possible to distinguish after recent fractures between cases in which perfectly satisfactory callus has formed, and cases which will go on to non-union. Neither can fibrous union be distinguished from union by callus in which lime salts have not yet been deposited. 5. The evidence as to X-ray burns seems to show that in the majority of cases they are easily and certainly preventable. It seems not unlikely when the strange susceptibilities, due to idiosyncracy, are remembered, that in a small number of cases it may make a given individual especially liable to this form of injury. 6. In the recognition of foreign bodies the skiagram is of the very greatest value; in their localisation it has occasionally failed. Mistakes in localisation are becoming less frequent by the adoption of accurate mathematical methods, but the surgeon who bases an important operation on the localisation of a foreign body buried in the tissues, should remember the possibility of error that still exists.

Though the question of the influence of **oöphorectomy in cancer of the breast** cannot yet be finally decided, the subject must arrest our attention, when we review the results obtained by various operators. Mr. Stanley Boyd[1] and Dr. Ernest Herman[2] contributed two helpful papers on this matter at the recent meeting of the British Medical Association at Ipswich. Mr. Boyd has collected 54 cases, and divides them into two groups: 1. Those in which oöphorectomy seemed to produce a clear and considerable result. 2. Those in which oöphorectomy had little or no effect, or in which the effect was doubtful. Thus classified, he maintains that of the 54 cases, 19 (35 per cent.) were more or less markedly benefited, 34 were not benefited, or were only doubtfully benefited, and one died of exhaustion.

He considers that such a success cannot be due to chance, to laparotomy (as in the case of supposed malignant disease of the abdomen cured by exploratory laparotomy), or to the administration of thyroid extract.

Dr. Herman, however, considers that the administration of thyroid extract is important, and he points out that of 17 cases quoted by Mr. Boyd as being favourably influenced by oöphorectomy, 12 had been given thyroid extract; whereas, there were 21 cases in which no benefit had followed and only 5 of these had had thyroid.

Although, wasting of the cancer follows oöphorectomy in

[1] *Brit. M. J.*, 1900, ii. 1161. [2] *Ibid.*, 1167.

one case out of every three, it appears from Mr. Boyd's cases that cancer growths generally reappear or begin again to increase in six to twelve months.

This, however, means a gain of health and a prolonged life for a year or more to the patient. Six per cent. of the cases gained immunity for two years and over.

It appears that cancers other than mammary are not benefited by oöphorectomy, and that all mammary cases are not affected thereby. Mr. Boyd's theory is that certain ovaries, probably by pathological variation in their internal secretions, favour the growth of cancer by action, either upon the growth or upon the tissues; the removal of such ovaries alone will be of benefit though at present there is no clue to their recognition.

Returning to the two groups, the following points of difference are noted between the cases which improved and those which did not: 1. *Age.* The average age of group 1 is considerably higher than group 2; it has often been supposed that the cases in which oöphorectomy would be most likely to do good would be those in young women; this would seem not to be the case. 2. *Type of Case.* The cases in group 1 are of a more chronic type than those in group 2. 3. *Effect of menopause.* That with one exception all the patients who had passed the menopause are included among the failures. 4. *Effect of amount of disease.* The cases in group 2 are rather worse than those in group 1. The quantity of superficial disease does not, however, make the difference between failure and success; but whenever it has been evident that viscera or bones have been affected, no improvement in them has taken place. 5. *Time of appearance and course of improvement.* Pain often ceases at once, and may be permanently relieved. Vascularity and general fulness are diminishing and nodules are shrinking within a week. Much of the growth may have gone within two to four weeks. 6. *Effects of oöphorectomy.* Amenorrhœa and sterility, atrophy of the uterus and more slowly of the breasts are constant. Unpleasant effects seem to be rare. The following conclusions would therefore seem justifiable: Oöphorectomy should be offered in cases other than the very acute, in women over forty, with no visceral or bony lesions, in fair condition and before the menopause. In extensive primary cases and recurrences it seems advisable to remove what is possible of the disease; but healing should be attained, lest the oöphorectomy fail in its object and malignant ulceration be started.

The operation is not a severe one and in cases where the disease is so advanced that a speedy local recurrence seems likely, oöphorectomy might be performed at the same time that the local mischief is removed.

In most departments of life there is a tendency to follow each other without stopping to question the correctness of the views of our "leaders."

An honest scepticism should always set us thinking, and the revolutionary ideas on the **treatment of inguinal hernia** in childhood displayed by Mr. Russell[1] are worth consideration.

The popular view on this subject is that hernia in children may almost always be cured by a truss; and that operation is, therefore, rarely called for.

Mr. Russell entirely disagrees with this for he believes that all inguinal herniæ are accompanied by a congenital sac which no truss pressure can cure; and he deduces the following conclusions from his experience of operation in children. 1. Inguinal hernia in young subjects is caused by the presence of a congenital sac and there is no other cause. 2. The sac is in the vast majority of cases an unobliterated portion of the processus vaginalis. 3. Acquired oblique inguinal hernia in the young has no existence in fact. 4. Seeing that oblique inguinal hernia cannot occur in the absence of a congenital sac, there can be no recurrence after the sac has been efficiently removed.

The reason that he gives for stating that an acquired sac cannot occur is the existence of an automatic, self-regulating sphincter mechanism in the inguinal canal which he described in a former paper[2] as being formed by a rigid tendinous half, and an actively contracting muscular half. "Now when curved muscular fibres contract they straighten, and the effect of the straightening of these curved fibres is to cause them to descend towards a position parallel to the floor of the canal and so close the opening. In other words, the action of the curved fibres is sphincteric, and the original canal is a half sphincter and is closed, so far as the structures passing through it permit, by this sphincter action." The author is therefore of opinion that all cases of oblique hernia occurring at any time during the course of life take place in subjects who are the possessors of a congenital sac, and that subjects who have never possessed a congenital sac, or in whom the sac has been efficiently removed, can never become the subjects of oblique hernia.

In answer to the objection that herniæ frequently occur after operation, he maintains that such so-called "recurrences" are not true recurrences at all, but traumatic hernia due to misguided methods of operating. This is certainly a "facer" even for those who think that operations after the Bassini method or some modification of it are to be regarded as an over-doing of surgery except in the minority of cases.

James Swain.

[1] *Lancet*, 1900, ii. 1128. [2] *Ibid.*, 1899, ii. 1355.

